# Neurasthenia

**Published:** 1887-03

**Authors:** Reunette E. Fuller

**Affiliations:** Macon, Ga.


### NEURASTHENIA.*

BY MRS. REUNETTE E. FULLER, M. D.

The form of neurasthenia which I propose to make the subject of discussion I shall designate

### MENTAL DYSPEPSIA.

The average American is to a greater or less extent a confirmed dyspeptic. He complains of his stomach as the weakest part of his physical system. It is to him the heel of Achilles, where the arrows of pain and disease inflict the mortal wound. Like the bankrupt treasury of a nation, it not only ceases to afford a generous relief to a thousand legitimate demands, but it becomes a cause of pain, apprehension and torment.

The stomach supplies building material to the architectural edifice of the human body. It supplies material to repair the waste incidental to organic and intellectual life.

The nervous tissue must suffer waste in order that the current of thought may flow. Every thought, every logical process, is the product of certain chemical changes in the elements of the nervous tissue from the condition of organic life to that of dead matter.

Thought is a direct product of the disorganization of a portion of the human brain. The plane of thought as truly feeds upon the material substance of the brain as the smelting process in the reduction of ore consumes the glowing coal in the furnace.

In mental dyspepsia the stomach has ceased to furnish the brain with sufficient material to equal the waste caused by the intellectual effort. The successive steps by which the scholar is unconsciously led down a pathway of physical bankruptcy, having their origin in mental dyspepsia, are important and interesting from a physiological point of view. The relations existing

* Read before the Macon Medical Society.

between mental dyspepsia and intemperance are entirely overlooked by the physiological writer and teacher.

In the briefest possible manner I will attempt a description that thousands will recognize in their own personal experience. In a brief glance within so wide and important a field, only a few of its most important points can receive attention.

The nervous tissue is, without exception, the most important of any part of the body. Within the gray cells of this tissue the *spiritus vitalis* (or life force) is stored, that mysterious life-principle that awakens into activity all of the phenomena of vitality exhibited by the various organs of the body. Of course this tissue requires a constant supply of building material to compensate the waste that is constantly going on in its substance. Every thought of the brain, every contraction of a muscle and every secretory or excretory function of an organ requires in the act a certain expenditure of nerve-force or energy, and the production of this must necessarily require, in turn, the supply of nerve-food.

Now, an animal tissue has the habit, like the cannibal, of feeding upon its own substance, relying upon the stomach to replace the amount consumed as rapidly as it takes place. Let it always be borne in mind that the nervous tissue, in the elaboration and distribution of its own proper secretion—life-force—feeds upon itself, and also that it requires of this tissue to supply all the other tissues in the body, and at the same time the fountain of organic and rational life. From this it will be clearly understood that the labor performed by the nervous tissue is incomparably greater than any other in the human body.

Now, the amount of building material required by a living tissue to meet the waste resulting from labor is always in proportion to the amount of labor performed. Food is a latent form of vital action, and vital action is the source of labor. Hence, whenever it happens that the stomach is not supplied with proper and sufficient food to repair the waste of nervous tissue, almost infinite physiological mischief follows. The nervous tissue falters in its function, and the wheels and levers in the various dependent organs vibrate with a diminished speed, while the instinct of self-preservation becomes aroused as the starving nervous tissue through the sense of hunger clamors for food. Every other tissue, experiencing a deficiency in vital action, demands food. Like the voice of a multitude the cry goes up to the ear of reason—food! food! food!

The victim of this dietetic error is rationally conscious of a deficiency. His reason hears the voice of nature, but her language is unintelligible, or he does not wish to understand. Of course he is not a master of dietetic science. The victim devours with an almost insatiable appetite the fat and muscle-forming varieties of food that chiefly constitute the present popular bill of fare. At last his digestive organs falter in their function beneath the weight of the useless burden. His nature, intellectual and physical, feels crushed beneath an unsatisfied want, and even staggers and reels under the invisible burden. If the labor he is accustomed to perform is chiefly intellectual, the mischief and disaster to the whole system is doubly increased; for the process of intellection occasions a much greater proportional waste of the elements that compose its substance than is exhibited by the other portions of the nervous tissues in the performance of the purely organic processes. At this juncture the condition of the victim, from a physiological point of view, is painful in the extreme; for the stomach, in common with the other organs of the body, experiencing a deficiency in vital energy, besides being crushed beneath a fatal burden, signally fails to fulfill its important office of supplying the waste incidental to vital action, and the blood in consequence becomes still more impoverished in elements requisite to supply the demand of the nervous tissue. The germ of that uncomfortable disease, mental dyspesia, has taken root. The victim is tortured with a longing and craving that is almost insupportable. He feels depressed, languid and gloomy. A sense of weariness, which he expresses in the significant word "goneness," never forsakes him. He toils at his daily task, but he does it with a painful effort. He will not admit to his friends that he is sick, and he becomes ill-natured and indolent. An effort that he once would have made with assurance and delight now assumes proportions from which he recoils with fear and distrust. At last the torture becomes unbearable and he calls a physician. Then the crisis has come. Nine times out of ten, to "brace up his system" and to quiet his rebellious nerves, the medical practitioner prescribes a brain stimulant, either alcohol or opium, as the thing indicated as the proper remedy, or frequently combines them in a single prescription. A poison is given to supply the place of concrete, assimilable brain-food. Under its stimulating effects the patient feels invigorated. But the seeming salutary effect is founded upon the fatal physiological law that brain stimulants temporarily supply the place of brain and nerve-food. The dose of the poison from time to time is given more frequently and increased in quantity as the demand arises. The victim finds on experience that during the period of excitement resulting from its stimulating effect that he can resume his uninterrupted labor. But between the periods of excitement his mental and physical torture seems to be doubly aggravated. It may be that his daily toil earns his daily bread. If so he must work, even if he works under the excitement of a stimulating poison. But his nature, physical and intellectual, is being fettered by a chain whose invisible links are stronger than those of steel. In this manner the appetite for stimulating brain-poisons is innocently and unconciously acquired by the victim of mental dyspepsia.

In this path which I have just described thousands of gifted minds in our land are at this moment descending to shame, ruin and degradation to fill a drunkard's grave. Now, the question comes to every thinking mind: On whom does the guilt rest? I answer: First, upon society for permitting the dietetic error that makes the ruin possible; second, upon the physician who substitutes a poison for assimilable brain-food. The number of these victims is increasing from year to year in a frightful ratio.

After a little thought and reflection all will agree that intellectual activity has increased in our land for the past fifty years; that among a given number of population four times as many books and newspapers are read, four times as many subjects studied and mastered, four times as many opinions promulgated and defended in the place of a single one fifty years ago. Now, this being the case, what does it suggest to the thinker of to-day? To the physiologist it suggests a proportional increase in the present dietetic system of those elements of food that supply the waste of the nervous tissues arising from the intellectual process.

Now comes the query: has there been such an increase? I say *emphatically no*, and every chemist and physiologist will agree with me. As a result from the terrible friction incidental to social, professional, commercial and political life, an ill-fed cerebral tissue is the inevitable; hence, the average individual engaged in any occupation or profession requiring continuous and intense intellectual activity is usually broken down physically and intellectually between the ages of forty and fifty years. We see examples of these daily, and even worse. Now and then these conditions become so extreme that reason is dethroned. We cannot tell how soon that destroying bolt will descend. Incipient insanity may gradually come on, subdue the strongest will and cloud the brightest intellect long before the victim dreams of what is coming.

How many men and women become old at this age and give up their places for younger ones who are only too soon to follow in their footsteps! This condition to me seems to be a great loss of time—a failure in the end that might have been avoided if the foundation had been fitly laid, but instead he or she crawls away upon the shelf to die a physical and intellectual wreck.

The instinct of self-preservation demands to-day that every mind should seriously and anxiously ask itself the important question, am I in danger? If so, am I doing my duty toward remedying the evil? Show me a clouded intellect with a timid, vacillating, capricious will—that physical and mental condition which constitutes the incipient stage of insanity—and I will invariably show you in the daily bill of fare of this individual the presence of fat and muscle-forming elements of food and an almost entire absence of nerve-building staples. Mental dyspepsia as a disease is terrible; but to have it become the prison-walls of an abominable, fatal vice while the victim is unconscious of danger is, indeed, a horrible doom. Thousands have become slaves to stimulating brain-poisons through mental dyspepsia. Mental power and culture are royal prerogatives. In the minds of the masses they confer a kind of dignity and nobility even on an element of vice that may be associated with them in the same character. As a result, the scholarly or intellectual drunkard is to-day diffusing the vice of intemperance in every circle of human society, giving it a certain respectability and dignity derived from its association while commanding intellectual power. In this way this deadly vice is borne to take root in virgin soil which otherwise it had never reached.

*The remedy* for mental dyspepsia is suggested by its cause. It can be nothing else but natural assimilable brain-food joined to an efficient fulfillment of the digestive function. This food must contain in itself the same chemical elements that enter into the constitution of the nervous tissue. It must be concrete, soluble with chemical affinities so easily broken as to impose the least possible tax upon the digestive organs. To this we must add an observance of physical laws relating to the functions of digestion and assimilation.

---

IODOFORM AND SILVER.—A curious effect of iodoform upon silver is reported by Dr. Poncet, who had his attention directed to it by a patient, to whom he had been applying iodoform dressings, telling him that all soups and other diet for which she used a silver spoon had a very disagreeable taste, and the spoon itself had a garlicky odor. Upon investigating the subject Dr. Poncet found that silver that has been in contact with iodoform, or which is even touched by the fingers after they have been in contact with iodoform, acquires a nauseous odor, resembling that of garlic, which becomes more perceptible upon rubbing the silver. A drop of saliva from a patient fully under the influence of iodoform is said to be sufficient to impart this odor to silver, or the mere exposure of iodoform and silver in the neighborhood of one another. The odor is not that of iodoform, but is thought to be due to a decomposition product.—*Medical Press and Circular, January 19, 1887.*

